# Neurodegeneration-Associated Proteins in Human Olfactory Neurons Collected by Nasal Brushing

**DOI:** 10.3389/fnins.2020.00145

**Published:** 2020-03-05

**Authors:** Lorenzo Brozzetti, Luca Sacchetto, Maria Paola Cecchini, Anna Avesani, Daniela Perra, Matilde Bongianni, Corinne Portioli, Maria Scupoli, Bernardino Ghetti, Salvatore Monaco, Mario Buffelli, Gianluigi Zanusso

**Affiliations:** ^1^Neuropathology Section, Department of Neurosciences, Biomedicine, and Movement Sciences, University of Verona, Verona, Italy; ^2^Otolaryngology Section, Department of Surgery, Dentistry, Paediatrics and Gynaecology, University of Verona, Verona, Italy; ^3^Anatomy and Histology Section, Department of Neurosciences, Biomedicine, and Movement Sciences, University of Verona, Verona, Italy; ^4^Physiology Section, Department of Neurosciences, Biomedicine, and Movement Sciences, University of Verona, Verona, Italy; ^5^Biology and Genetics Section, Department of Neurosciences, Biomedicine, and Movement Sciences, University of Verona, Verona, Italy; ^6^Department of Pathology and Laboratory Medicine, Indiana University School of Medicine, Indianapolis, IN, United States

**Keywords:** olfactory neurons, olfactory neuroepithelium, olfactory brushing, neurodegenerative diseases, misfolded proteins

## Abstract

The olfactory neuroepithelium is located in the upper vault of the nasal cavity, lying on the olfactory cleft and projecting into the dorsal portion of the superior and middle turbinates beyond the mid-portion of the nasal septum. It is composed of a variety of cell types including olfactory sensory neurons, supporting glial-like cells, microvillar cells, and basal stem cells. The cells of the neuroepithelium are often intermingled with respiratory and metaplastic epithelial cells. Olfactory neurons undergo a constant self-renewal in the timespan of 2–3 months; they are directly exposed to the external environment, and thus they are vulnerable to physical and chemical injuries. The latter might induce metabolic perturbations and ultimately be the cause of cell death. However, the lifespan of olfactory neurons is biologically programmed, and for this reason, these cells have an accelerated metabolic cycle leading to an irreversible apoptosis. These characteristics make these cells suitable for research related to nerve cell degeneration and aging. Recent studies have shown that a non-invasive and painless olfactory brushing procedure allows an efficient sampling from the olfactory neuroepithelium. This approach allows to detect the pathologic prion protein in patients with sporadic Creutzfeldt–Jakob disease, using the real-time quaking-induced conversion assay. Investigating the expression of all the proteins associated to neurodegeneration in the cells of the olfactory mucosa is a novel approach toward understanding the pathogenesis of human neurodegenerative diseases. Our aim was to investigate the expression of α-synuclein, β-amyloid, tau, and TDP-43 in the olfactory neurons of normal subjects. We showed that these proteins that are involved in neurodegenerative diseases are expressed in olfactory neurons. These findings raise the question on whether a relationship exists between the mechanisms of protein aggregation that occur in the olfactory bulb during the early stage of the neurodegenerative process and the protein misfolding occurring in the olfactory neuroepithelium.

## Introduction

Olfactory brushing is a novel and non-invasive procedure for sampling neurons of the olfactory mucosa (OM). OM sampling is useful for the *in vivo* diagnosis of human prion diseases. In fact, OM samples obtained from patients with Creutzfeldt–Jakob disease and tested using the real-time quaking-induced conversion (RT-QuIC) assay provides a diagnostic accuracy of nearly 100% (Orrú et al., 2014; Zanusso et al., 2016). The amount of cells collected by a single sampling was one million of the total cells, and around 30% of them were immunopositive for the olfactory marker protein (OMP) (Orrú et al., 2014).

Olfactory mucosa is composed of neural cells; these originate from stem neural cells, which mature as glial or neuronal cells under the influence of specific growth factors. The pseudostratified neuroepithelium is made up of olfactory sensory neurons (ONs), supporting cells, microvillar cells, basal stem cells, and Bowman’s gland ductal components. The underlying connective *lamina propria* includes Bowman’s gland bodies, axonal fibers, and blood vessels (Welge-Lussen and Hummel, 2014). Olfactory neurons have an average lifespan of approximately 60 days (Sultan-Styne et al., 2009; Brann and Firestein, 2014) and are constantly replaced by younger neurons deriving from basal stem cells.

The ONs are slender bipolar cells with modified, non-motile sensory cilia, which have a primary role in the olfactory transduction process. ON axons, passing through the cribriform plate, project to the olfactory bulb, which is the first relay of the olfactory information. The second-order neurons (mitral and tufted cells) project to different olfactory areas (i.e., anterior olfactory nucleus, pyriform cortex, amygdala, and entorhinal cortex). The olfactory information spreads to associated neocortical areas involved in higher-order information processing (e.g., orbitofrontal cortex).

In the healthy aging population, olfactory dysfunction is commonly due to different causes such as multiple damages to the olfactory epithelium by microbial and/or environmental factors, age-related epithelial atrophy, and ossification of the foramina of the cribriform plate (Doty et al., 2014; Pinto et al., 2015). However, recent studies showed that an impaired olfactory function might predict a cognitive decline associated with a subclinical neurodegenerative process among older adults (Dintica et al., 2019). In most neurodegenerative diseases, hyposmia or anosmia occurs long before the onset of clinical signs. In patients with Parkinson’s disease (PD), the olfactory impairment might precede the motor dysfunction by many years (Haehner et al., 2007; Doty, 2012) and correlates with a decline in cognition (Cecchini et al., 2016, 2019; Iannilli et al., 2017; Masala et al., 2018). Furthermore, in Alzheimer disease (AD), the extent of olfactory dysfunction might predict the conversion of mild cognitive impairment to AD (Devanand et al., 2008). Thus, in the aforementioned conditions, olfactory deficit might be considered a prodromal symptom of neurodegeneration.

Several neuropathologic studies showed that the aggregation and deposition of proteins, such as α-synuclein, β-amyloid, hyperphosphorylated tau, and transactive response DNA-binding protein 43 (TDP-43) may occur within different parts of the olfactory system (Rey et al., 2018). In healthy individuals, protein misfolding may occur early in life, and chronic exposure to air pollutants might accelerate the protein aggregation. It has been also suggested that AD and PD pathology may occur in the olfactory bulbs as a result of inhalation of air pollutants (Calderón-Garcidueñas et al., 2019).

All together, the above data support the concept that ONs may serve as an ideal model for the analysis of early molecular stages of neurodegeneration. Earlier morphological studies of human OM were carried out using preparations of bioptic or autoptic mucosa (Morrison and Costanzo, 1990; Talamo et al., 1991; Trojanowski et al., 1991; Paik et al., 1992; Lee et al., 1993; Brouillard et al., 1994; Crino et al., 1995; Feron et al., 1998; Witt et al., 2009; Hummel et al., 2010; Holbrook et al., 2011; Funabe et al., 2013; Tanos et al., 2017). However, inherent to the technique of nasal biopsy, there are several limitations, for example, invasiveness, technical difficulties, and medical complications. Thus, to harvest ONs, we have used the technique of olfactory brushing, a harmless and non-invasive procedure. Using this approach, we are able to bypass the potential complications of a biopsy procedure.

The aim of the present study is to characterize specific phenotypic markers of the human olfactory cells by immunocytochemistry, which is the detection of the OMP, β-tubulin III (TUJ-1), protein gene product 9.5 (PGP 9.5), and the cytokeratins. Furthermore, with the aid of these markers, we determined the expression pattern of the following proteins, well known to be involved in neurodegenerative diseases: α-synuclein, β-amyloid, tau, and TDP-43.

To our knowledge, the present study is the first to investigate, in living healthy young and elderly human subjects, the phenotypes of primary ONs, relative to the specific proteins that may be involved in the neurodegenerative cascade, during aging, and in dementia.

## Materials and Methods

### Recruitment and Eligibility

Thirty healthy volunteers underwent nasal swabbing. These included 15 males (mean age: 50.4 years; range: 22–78 years) and 15 females (mean age: 54.2 years; age range: 19–79 years). Exclusion criteria were the presence of pathologies affecting the olfactory function (e.g., recent head trauma, rhinitis or chronic sinus infection, diabetes, stroke, history of smoking, and alcohol consumption). Olfactory brushing was performed following the approval of the ethical committee of the University Hospital of Verona (Prot. n. 28917, June 15, 2012). OM sampling was performed after each subject gave a written informed consent.

### Olfactory Brushing and Immunocytochemistry Procedures

Olfactory mucosa samples were obtained by nasal brushing, as reported (Orrú et al., 2014; Bongianni et al., 2017). Briefly, following nasal inspection using a rigid endoscope, olfactory cells were collected by means of a specifically designed flocked nasal brush (FLOQBrush^TM^, Copan Italia Spa, Brescia, Italy).

After sampling, the swab was immediately immersed in a 15-ml Falcon tube containing fixative solution (Diacyte, Diapath, Italy). A brief treatment with mucolytic CytoRich Red (Diapath S.p.A., Italy) was carried out to solubilize the proteins.

The cellular pellet was washed by serial passages in phosphate-buffered saline solution (PBS) and the cell suspension cytocentrifuged (CYTOSPIN IV, AHSI, Italy) onto microscope slides. Slides were preincubated for 1 h in a blocking solution (5% of normal serum of the same animal species of secondary antibody generation, 0.3% of Triton X-100 in 0.1 MPBS). Primary antibodies (listed in Tables 1, 2) were diluted in blocking solution and incubated overnight at 4°C. After three washings of 5 min each, goat anti-mouse and goat anti-rabbit or donkey anti-goat and donkey anti-rabbit secondary antibodies Alexa Fluor-conjugated (diluted 1:1,000; Life Technologies, Carlsbad, CA, United States) were incubated for 1 h at room temperature. Nuclear DAPI counterstain (1:2,000) at 405-nm emission wavelength was supplied directly before mounting the slides with ProLong Antifade Mountants for fixed cells (Thermo Fisher Scientific Inc., Italy). The non-specific immunostaining of secondary antibodies was controlled in each immunostaining session by omitting the first antibody. Slides were observed at confocal inverted Leica TCS SP5 AOBS microscope using 40× and 63× oil immersion objectives (1.25 NA). Images were saved as tiff files; brightness and contrast were adjusted with the Leica Application Suite Advanced Fluorescence (*LAS AF*) Software (Leica Mycrosystems, Wetzlar, Germany), with ImageJ (NIH, Bethesda, MD, United States) or ImarisX64 7.2.1 (Bitplane AG, Zurich, Switzerland).

**TABLE 1:** Primary antibodies used for immunophenotypic characterization of olfactory brushing samples.

**Antibody**	**Host**	**Antigen**	**Code (clone)**	**Company**	**Working dilution**
Anti-OMP	Rabbit polyclonal	Olfactory marker protein	sc-67219 (FL-163)	Santa Cruz	1:400
Anti-OMP	Mouse monoclonal	Olfactory marker protein	sc-365818 (B-6)	Santa Cruz	1:400
Anti-OMP	Goat polyclonal	Olfactory marker protein	544-10001-WAKO	Wako	1:400
Anti-β-tubulin III	Rabbit polyclonal	aa residues 441–450 of β-tubulin class III	T2200 (TUJ-1)	Sigma-Aldrich	1:400
Pan-cytokeratin	Mouse monoclonal	All isoforms of cytokeratin protein	MA5-15507	Thermo Fisher Scientific	1:300
PGP 9.5	Rabbit polyclonal	Ubiquitin carboxy-terminal hydrolase L1	Z5116	DAKO	1:300

**TABLE 2:** Primary antibodies used for characterizing the expression of distinct neurodegeneration-associated proteins.

**Antibody**	**Host**	**Antigen**	**Code (clone)**	**Company**	**Working dilution**
Anti-α-synuclein	Mouse monoclonal	Full-length human α-synuclein	ab1903 (4D6)	Abcam	1:750
Anti-APP	Mouse monoclonal	aa residues 1–16 of β-amyloid precursor protein	SIG-39320 (6E10)	Covance	1:200
Anti-tau 5	Mouse monoclonal	Total microtubule-associated protein tau	AHB0042 (tau-5)	Thermo Fisher Scientific	1:500
Anti-4R tau	Rabbit monoclonal	aa residues 250–350 of 4R isoform of tau	ab218314 (EPR21725)	Abcam	1:500
Anti-3R tau	Mouse monoclonal	aa residues 209–224 residues of 3R isoform of tau	05-803 (8E6/C11)	Merck Millipore	1:500
Anti-TDP 43	Rabbit polyclonal	aa residues 1 to 260 of TAR DNA-binding protein 43	10782-2-AP	Proteintech	1:200

## Results

The immunocytochemical characterization was carried out in OM samples obtained from all the subjects included in the study. Although inter- and intra-subject variability was observed in the number of collected cells, the quality of immunocytochemical pattern was identical and reproducible in all samples analyzed. This evidence was consistent relative to the expression patterns of the specific ON proteins as well as for α-synuclein, β-protein, tau, and TDP-43.

### Phenotypic Characterization of the Epithelial Cellular Samples

**FIGURE 1:**
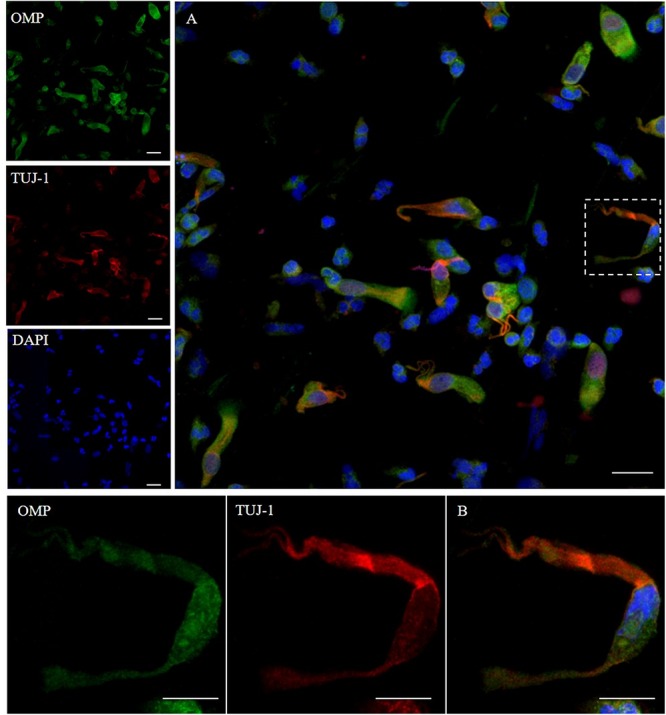
Pattern of immunolabeling by olfactory marker protein (OMP) and β-tubulin III (TUJ-1) in cell obtained by olfactory brushing. Sample harvested by olfactory brushing and analyzed following centrifugation. Double immunostaining with OMP (green) and TUJ-1 (red). Several cells with different morphology showed a cytosolic positivity to OMP. Most of round cells and non-neural-shaped cells show an intense positivity in the cytoplasm, while olfactory neurons show a faint granular pattern. In contrast, TUJ-1 stains mainly the neural processes, in particular, the axonal hillock of cells, identified as olfactory neurons [outlined square in **(A)** details in **(B)**]. Weak cytosolic positivity is also observed in the apical portion of supporting-like cells. Scale bar **(A)**: 20 μm. Scale bar **(B)**: 10 μm.

To ensure that the cellular collection was done on the olfactory area, we first assessed the cellular expression of OMP and neuron-specific class III β-tubulin. OMP immunoreactivity was mainly intracytoplasmic with a homogenous distribution array, while TUJ-1 showed a main axonal pattern tracking the extension of the neural process emerging from the axonal hillock (Figure 1). Further, non-neuronal-shaped cells, most likely the supporting cells, showed positivity to the OMP antibody.

**FIGURE 2:**
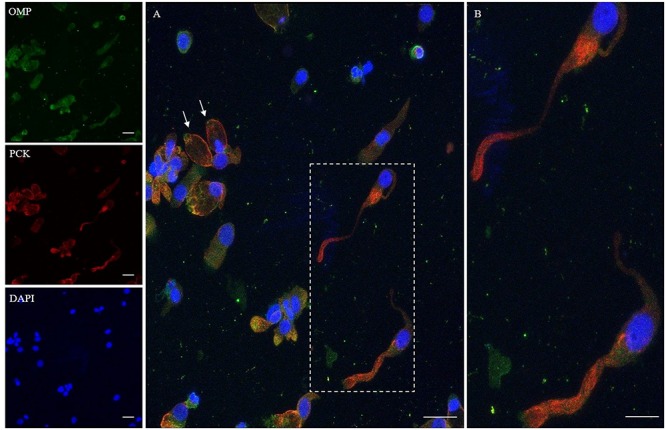
Immunocytochemical analysis of a cytocentrifuged sample of olfactory mucosa (OM) using OMP (green) and PCK (red). While OMP stains round and non-neuronal-shaped cells, PCK preferentially stains the whole apical dendritic projection of olfactory neurons [outlined square (**A**) up to the cilia boundary (detail **B**)]. Interestingly, in ONs, the immunopositivity with PCK is distributed on the opposite side of that obtained with β-tubulin III. In the other cells, PCK expression is distributed on the boundary of the cell body, all along the plasma membrane (arrows) of cells that have a round shape. Scale bar **(A)**: 20 μm. Scale bar **(B)**: 10 μm.

**FIGURE 3:**
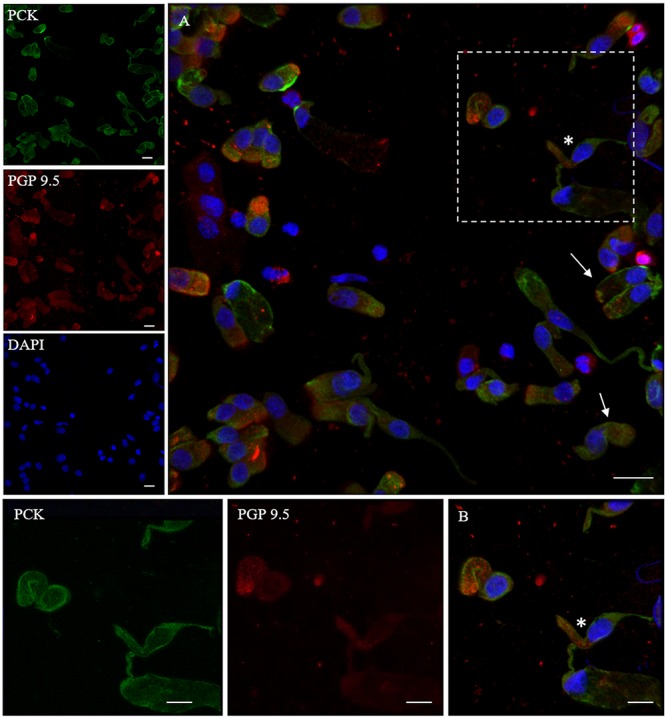
Distribution pattern of PGP 9.5 and PCK. PGP 9.5 (red) shows a dotted positivity mostly in the cytosol of cells with sustentacular-like morphology and less intensely in olfactory neurons (*, square detail). PCK (green) positivity is also identified in the cytosolic compartment of olfactory neurons and on the plasma membrane of other cells with non-neuronal morphology (arrows). Scale bar **(A)**: 20 μm. Scale bar **(B)**: 10 μm.

In addition, we examined the expression of pan-cytokeratin (PCK), a typical epithelial marker, and that of the PGP 9.5, a cytoplasmic protein in neurons and neuroendocrine cells. PCK antibody revealed a positive signal in all the epithelial cells (Figure 2). In neuronal-shaped cells, PCK was more intense in the cytosolic compartment, and the main positivity was observed on the superior dendritic projection. In contrast, non-neuronal-shaped cells showed more evident staining on the plasma membrane (Figure 2). The sample characterization had been last achieved by the combination of both PCK and PGP 9.5 markers (Figure 3). PGP 9.5 positivity sporadically revealed a coin-shaped staining pattern in the cytosolic apical pole of supporting cells (Figure 3). The morphology of these large and columnar cells is different from the thin and fused-shaped ONs whose apical part clearly shows the dendritic knob. A few neuronal-shaped cells showed a less intense PGP 9.5 positivity (Figure 3B, asterisk).

### Neurodegeneration-Associated Protein Expression Pattern

#### α-Synuclein

**FIGURE 4:**
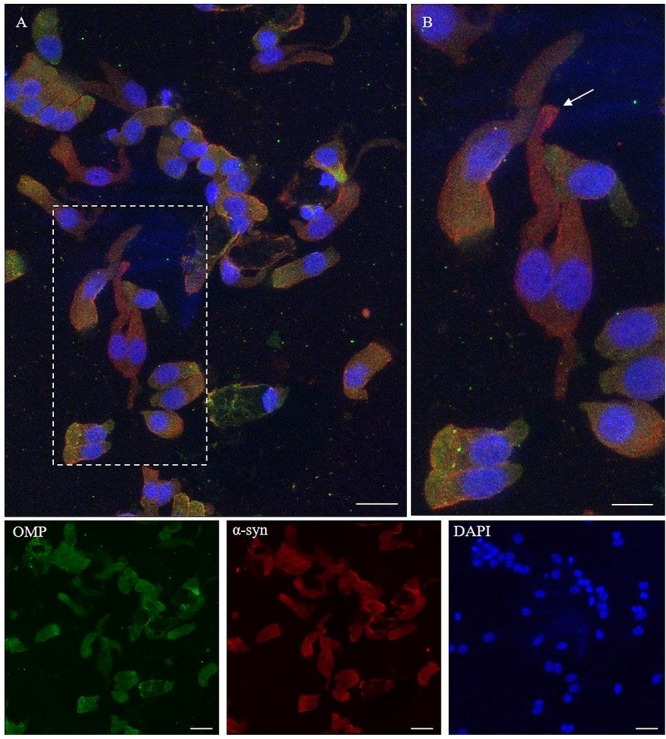
Immunocytochemical pattern of α-synuclein distribution in OM samples. Alpha-synuclein (4D6, red) shows a diffuse cytosolic distribution as well as a granular labeling around the margin of the plasma membrane of olfactory neurons and non-neuronal-shaped cells. Particularly, olfactory neurons show a thin granular labeling particularly localized at the dendritic knob [outlined square in **(A)**, arrow in detail in **(B)**]. In the other cells, OMP-positive (green) α-synuclein shows a positivity around the plasmatic membrane. Scale bar **(A)**: 20 μm. Scale bar **(B)**: 10 μm.

The α-synuclein is an unfolded 140-amino acid protein encoded by the *SNCA* gene and with a function not completely known. The distribution of the protein is ubiquitous, but it is mainly expressed at the tips of neurons as a pre-synaptic protein. The expression of α-synuclein in the olfactory samples was determined by 4D6, a monoclonal antibody that, generated from the non-modified full length of α-synuclein, binds all the isoforms of the protein, regardless of the post-translational modification. As shown (Figure 4), the expression pattern of α-synuclein in non-neuronal-shaped cells was detected around the cellular membrane, while in neuronal-shaped cells, α-synuclein showed a predominantly granular positivity, which was visualized in the cytosol, in particular, at the level of the dendritic knob (Figure 4B, arrow).

#### β-Amyloid

*APP* gene encodes for the amyloid precursor protein, a transmembrane glycoprotein of 770 amino acids, which is processed through sequential cleavages performed by different secretases. The peptide β-amyloid derives from APP by sequential cleavages of β- and γ-secretase. APP is widely expressed in human tissues with preferential expression in the central nervous system (Wang et al., 2017). The 6E10 mAb reacts to residues 1–16 of β-amyloid.

**FIGURE 5:**
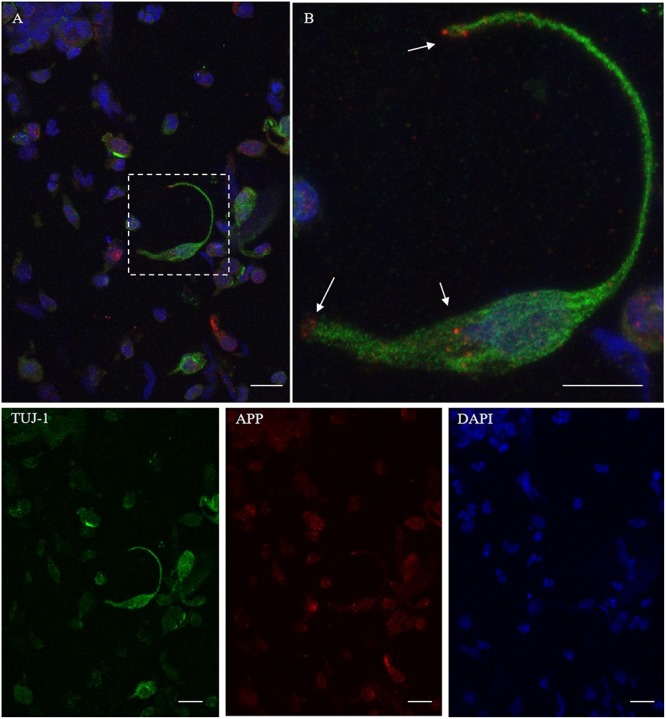
Distribution pattern of β-amyloid. Double immunostaining with TUJ-1 (green) and mAb to β-amyloid (red). Beta-amyloid shows a focal expression (dot-like) in the proximity of the nucleus and at the level of the surface tips of the olfactory neurons [outlined square in **(A)**, details in **(B)**, arrows]. Scale bar **(A)**: 20 μm. Scale bar **(B)**: 10 μm.

We found that the 6E10 immunoreacted with TUJ-1-positive ONs showing a dot-like distribution, around the nucleus and at the level of the surface tip of the cell (Figures 5A,B, arrows). Some non-neuronal-shaped cells show a faint positivity.

#### Tau

Tau is a microtubule-associated protein (MAP) and its function is that of binding to the microtubules and stabilizing them. In the adult human brain, six tau isoforms are generated from *MAPT*, the tau gene, through alternative messenger RNA (mRNA) splicing. Alternative splicing of exon 10 gives rise to three isoforms with three microtubule-binding repeats (3R) each and three isoforms with four microtubule-binding repeats (4R) each (Goedert et al., 1989).

**FIGURE 6:**
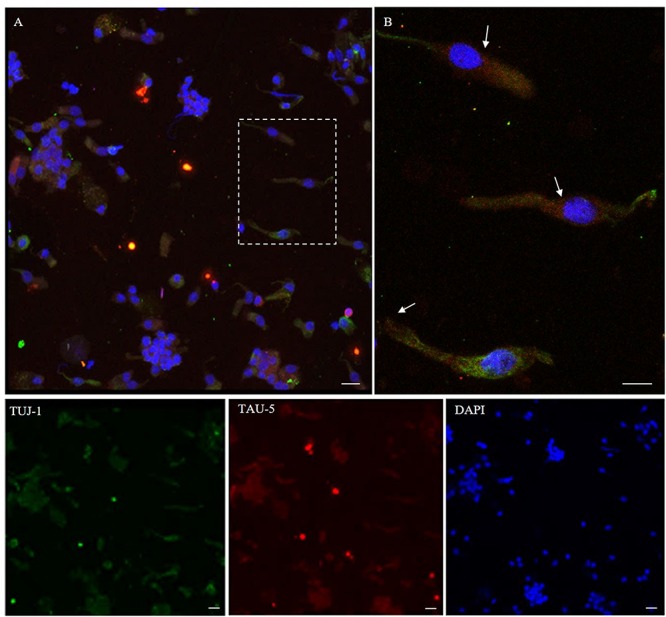
Distribution pattern of tau-5. Tau protein (tau-5) expression (red) is mainly detected in the cytosol of β-tubulin III-positive cells (green). Tau-5 immunopositivity is distributed along the neuronal body with particular intensity in the perinuclear region [outlined square in **(A)**; detail in **(B)**, arrows]. Scale bar **(A)**: 20 μm. Scale bar **(B)**: 10 μm.

**FIGURE 7:**
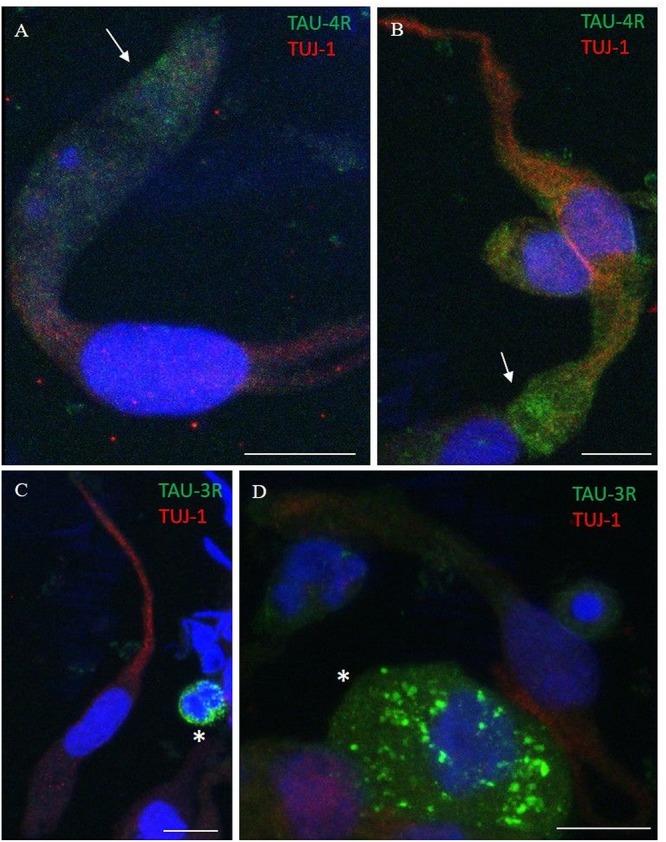
Distribution pattern of 4R and 3R tau isoform expression. Double immunostaining with TUJ-1 (red) and mAb to 4R tau isoform (**A**,**B**, green) and 3R tau isoform (**C**,**D**, green). **(A,B)** The 4R tau isoform (green) is mainly localized in the cytosolic apical portion of TUJ-1-positive olfactory neurons, (red) in the proximity of the dendritic knob (arrows). As opposite, TUJ-1-positive cells are negative to the 3R tau isoform (green), which is unevenly distributed as granules in rounded cells, likely in differentiated stem cells, negative for the TUJ-1 antibody (**C**,**D**, *). All scale bars: 10 μm.

Using immunostaining, total tau mAb (tau-5) was detected in the neuronal-shaped cells TUJ-1 positive with a cytosolic localization (Figure 6). The tau expression pattern was intracellular with a patched distribution along the cell body. Conversely, using antibodies to distinct tau isoforms, a positivity for 4R tau isoform in olfactory neurons was observed. This was remarkable within the area underlying the olfactory knob (Figures 7A,B, arrows). In contrast, 3R tau expression was unevenly clustered only in some rare round cells, which were likely to be basal stem cells (Figures 7C,D, asterisks).

#### T DP-43

**FIGURE 8:**
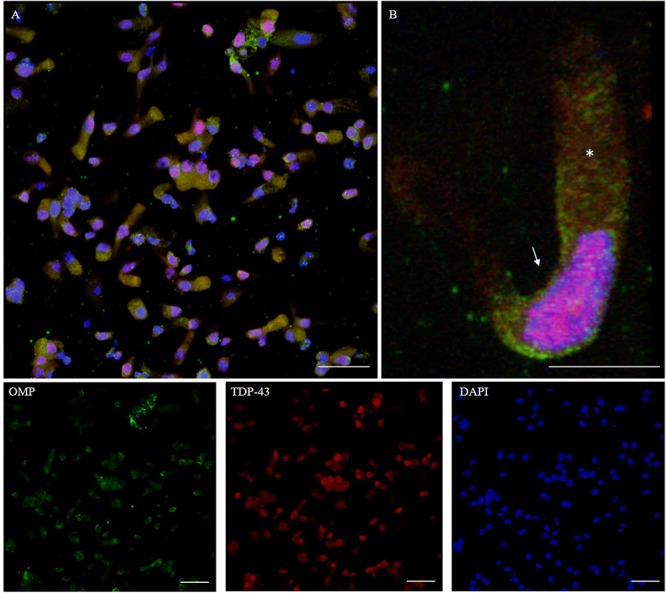
Distribution pattern of TDP-43. Double immunostaining of OM sample with OMP (green) and TDP-43 (red). TDP-43 shows granular staining in the nucleus (arrow) of almost all types of cells, albeit sparing rounded globular cells (**A**, detail in **B**). In particular, neuronal-shaped cells, OMP positive, shows an intense TDP-43 positivity in the nucleus and also a weak staining in the cytosol (*). Scale bar **(A)**: 40 μm. Scale bar **(B)**: 10 μm.

TDP-43 is a protein involved in the regulation of RNA processing. TDP-43 plays a role in transcription, alternative splicing, and mRNA stability. It is involved in various cellular processes, including apoptosis, cell division, and axonal transport. It is reported that in addition to being expressed in neurons, TDP-43 is abundantly expressed also in glia, as well as in many other cell types (Kawakami et al., 2019). TDP-43 immunostaining was detected in the nucleus. The intranuclear distribution of TDP-43 was intense; however, the immunolabeling revealed also weaker positive grains in the cytosol around the nucleus (Figure 8).

## Discussion

Olfactory impairment is recognized as a prodromal symptom in patients with neurodegenerative diseases. Thus, the olfactory system, and particularly the OM, may be considered as a peripheral neural tissue sentinel that allows exploring the trajectory of neurodegeneration *in vivo* and its pathogenesis.

In the present study, we showed that the human olfactory neuroepithelium might be easily sampled by the olfactory brushing technique. Through the latter, it is easy to collect ONs and other cells from neuronal lineage that can be used for cytological and immunocytochemical investigations. Olfactory brushing allows a gentle collection of OM, is easily performed, non-invasive, non-traumatic, and painless. The collected cells show a well-preserved morphology, enabling the recognition of ONs, which are characterized by a typical bipolar slender shape and are different from other harvested cellular components. In addition, it is noteworthy that the characterization of the cell components of the OM in humans and in animal models is important because it may provide novel insights about interspecies differences (Hodge et al., 2019). Since the types of harvested cells in each sample may vary, and the cell number may not be sufficient for the identification of cell types by morphological criteria, the first aim of our study focused on the search of specific neuronal markers that characterize the cytologic immunophenotype of the ONs; such markers would allow a distinction between ONs and other cellular components.

The following three important results (A, B, and C) were obtained from our studies directed to the first aim. (A) We confirmed that TUJ-1 directed to anti-class III β-tubulin is a reliable marker to identify ONs, as previously reported by studies of biopsy and autopsy tissue (Holbrook et al., 2011; Tanos et al., 2017). (B) We showed that PGP 9.5, a neuronal-specific marker, labeled ONs. Punctate staining was detected in the cytoplasm. This pattern appears to be different in other ciliated and tubular cells that showed a round staining in the cytoplasm. This labeling pattern confirms previous results obtained in human tissue (Johnson et al., 1997; Witt et al., 2009; Holbrook et al., 2011). These showed that both β-tubulin IV, a marker of the respiratory epithelium, and PGP 9.5 labeled non-neuronal cells. However, it cannot be ruled out that these cells that do not have a neuronal-shape but are found to be PGP 9.5 positive might be immature neurons or isolated metaplastic ONs. (C) Polyclonal antibodies directed to the OMP-labeled cytosol of ONs as previously reported (Witt et al., 2009; Holbrook et al., 2011); however, non-neuronal-shaped cells were also immunolabeled by the same antibodies. Although the physiological roles of OMP are not fully understood (Nakashima et al., 2019), it is widely accepted as a marker of mature ONs, even though OMP is also detectable in some non-olfactory tissues that are not classified as classical chemosensory (Kang et al., 2015).

Since our OM samples were obtained from the middle turbinate, we cannot exclude that OMP expression in some cells that do not have neuronal morphology might be related to a modulatory paracrine activity of these cells for the olfactory function of the ONs. In the middle turbinate, ONs are unevenly distributed, compared to the epithelium covering the cribriform plate in which neurons are densely present. Furthermore, in humans, the boundary of the olfactory epithelium is not sharply demarcated from that of the non-olfactory tissue, in contrast to rodents where the boundary appears to be well defined (Welge-Lussen and Hummel, 2014). In addition, in the human airway system, it was shown that ciliated cells have chemosensory features (Shah et al., 2009; Merigo et al., 2012), and as reported recently in the rat trachea, it is possible that the various epithelial cell populations having different chemosensory properties work together as a complex cellular network (Lasconi et al., 2019). Considering the dynamic properties of the olfactory epithelium, the OMP immunolabeling in non-neuronal-shaped cells might also suggest that these cells have yet to complete their maturation process. Indeed, in harvesting cells from a heterogeneous tissue, the olfactory brushing affects the architectural integrity of the epithelial surface. Therefore, recognizing the stages of neuronal maturation in cells that have been separated from the original environment may be challenging.

As a second aim of the present study, the expression pattern of proteins involved in neurodegenerative diseases was analyzed in human ONs of healthy individuals. Limited information is available on this aspect of the biology of this cell group. Studies carried out in rodents and in human tissue obtained at autopsy or by biopsy are available at this time (Rey et al., 2018). Indeed, in some neurodegenerative disorders such as AD or PD, different studies have shown the presence of neurofibrillary tangles, β-amyloid deposits, or Lewy neurites in the ONs (Talamo et al., 1989, 1991; Trojanowski et al., 1991; Lee et al., 1993; Crino et al., 1995; Funabe et al., 2013; Saito et al., 2016). These findings might suggest the hypothesis that abnormally conformed proteins may be transported from ONs to the glomeruli of the olfactory bulb where they accumulate, aggregate, and assemble into fibrils. In ONs, it might be difficult to observe the pathological changes typically seen in the neurons of the brain, since ONs undergo a complete cycle every 3 months, and this short time may be insufficient for visualizing mature aggregates. Thus, the step of demonstrating the expression of neurodegeneration-associated proteins in ONs from healthy subjects has significant implications.

We showed that ONs constitutively express proteins involved in neurodegenerative diseases; however, we observed that α-synuclein and TDP-43 can be detected not exclusively in ONs but also in other cells lacking the shape of neurons.

At present, a few studies defined the normal expression pattern of α-synuclein in human olfactory brain regions (Rey et al., 2018). However, in elderly healthy subjects, high levels of α-synuclein were seen in olfactory brain areas (Freer et al., 2016). Furthermore, it has been shown that α-synuclein is expressed in human OM and, specifically in ONs, supporting cells and Bowman’s gland component (Duda et al., 1999). Our results are consistent with the previous findings.

TDP-43 is widely present in nuclei, particularly in ONs; however, it appears to be also present in supporting cells. Indeed, this protein has been reported to be abundantly expressed in both neurons and glia (Gao et al., 2018). Two previous studies investigated TDP-43 in the olfactory system. The first study showed immunopositive inclusions in autopsy specimens of olfactory bulb and primary olfactory cortex of patients that had amyotrophic lateral sclerosis (ALS) and also olfactory dysfunction (Takeda et al., 2015). A second autopsy study, in a patient with ALS and olfactory dysfunction, showed TDP-43 immunopositive inclusions in the lower motoneurons and in neurons of the limbic system (Takeda et al., 2014).

Beta-amyloid revealed a dot-like pattern in the nerve cell terminals and in the cytoplasm of the neuronal perikarya. There are no data available relative to the β-amyloid expression in ONs in healthy humans; however, in the rat, it has been reported in the olfactory bulb and in cortical olfactory regions (Rey et al., 2018).

Tau protein expression had not been studied in olfactory structures in humans (Rey et al., 2018). The olfactory regions of rats revealed that tau is strongly expressed in ONs and in their axons within the olfactory bulb (Viereck et al., 1989; Schoenfeld and Obar, 1994). In the present study, we showed that tau immunolabeling was present around the nuclear compartment and in the apical region of the ONs; 4R tau immunopositivity was found under the knob, while 3R tau immunopositivity was absent. Conversely, 3R tau was detectable in some basal stem cells.

In conclusion, the evidence of constitutive expression, in normal human OM, of those proteins that become misfolded as neurodegenerative processes occur offers a promising new research direction. Since the olfactory neuroepithelium, including the ONs and neighboring supporting cells, is highly exposed to microbial, viral, and toxic/environmental insults, it is conceivable that such events might have some role in disrupting the physiological interaction of different cell types, potentially leading to olfactory signal impairment and even to protein misfolding. Thus, if protein misfolding occurs, oligomers or complex assemblies may also form; then, aggregates might be transported through the axonal anterograde pathway to the olfactory bulb where they can assemble into fibrillary structures and β–amyloid deposits. Research on the main pathological conditions, in which the aforementioned proteins are involved, is needed to further understand the role of ONs and supporting cells in neurodegenerative human diseases.

## Data Availability Statement

The datasets generated for this study are available on request to the corresponding author.

## Ethics Statement

The studies involving human participants were reviewed and approved by Ethical Committee of University Hospital of Verona (CESC). The patients/participants provided their written informed consent to participate in this study.

## Author Contributions

GZ, LB, and MBo contributed to the conception or design of the work. LB, MBu, MPC, and GZ contributed to the acquisition, analysis, and interpretation of data for the work. MPC, AA, DP, MBu, CP, and BG contributed to the data interpretation. GZ, LB, BG, and MPC contributed to writing the manuscript. GZ, LB, SM, MBo, LS, and MPC contributed to revising it critically for important intellectual content. LS contributed to performing the olfactory brushing. MS, SM, and MBo provided the laboratory equipments. All authors provided approval for publication of the final content.

## Conflict of Interest

The authors declare that the research was conducted in the absence of any commercial or financial relationships that could be construed as a potential conflict of interest.
